# The Microbiome, an Important Factor That Is Easily Overlooked in Male Infertility

**DOI:** 10.3389/fmicb.2022.831272

**Published:** 2022-03-02

**Authors:** Hefeng Wang, Anran Xu, Liping Gong, Zhaowen Chen, Bin Zhang, Xiuyun Li

**Affiliations:** ^1^Department of Pediatric Surgery, Shandong Provincial Maternal and Child Health Care Hospital, Jinan, China; ^2^Reproductive Medicine Center, Shandong Provincial Maternal and Child Health Care Hospital, Jinan, China; ^3^Department of Obstetrics and Gynecology, Yicheng Street Community Health Service Center, Linyi, China; ^4^Department of Obstetrics, Shandong Provincial Maternal and Child Health Care Hospital, Jinan, China; ^5^Department of Ophthalmology, Shandong Provincial Maternal and Child Health Care Hospital, Jinan, China; ^6^Key Laboratory of Birth Regulation and Control Technology of National Health Commission of China, Shandong Provincial Maternal and Child Health Care Hospital, Jinan, China

**Keywords:** microbiome, male infertility, microbial infection, microbial dysbiosis, antimicrobial agents, probiotics

## Abstract

Humankind has been interested in reproduction for millennia. Infertility, in which male factors contribute to approximately 50%, is estimated to concern over 72 million people worldwide. Despite advances in the diagnosis, medical treatment, and psychosocial management of male infertility over the past few decades, approximately 30% of male infertility is still thought to be idiopathic. Despite emerging advances in the microbiome associated with male infertility have indicated that the microbiome may be a key factor to the management of male infertility, roles, and mechanisms of the microbiome remain ambiguous. Here, we mainly discussed the association between microbial infection in the genital tract and male infertility, effect of antimicrobial therapy on male reproduction, association between microbial dysbiosis and male infertility, and effect of probiotic intervention on male reproduction. This review made progress toward establishing a relationship between the microbiome and male infertility, and explored the role of the microbiome in male infertility. We call for more high-quality studies to focus on the relationship between microbes and male infertility, and strongly suggest increasing awareness among sterile males with microbial infection and/or microbial dysbiosis when they seek fertility help.

## Introduction

Infertility is defined as couples who have had regular sex for more than 1 year without contraception failing to conceive and has been regarded as a major global public health issue. Infertility constitutes an emotional, social, and financial burden, yet appropriate services directed toward preventing and addressing infertility are often inaccessible, unaffordable, or non-existent (Dierickx et al., 2021). Compared with female infertility, too little attention has been given to male infertility.

The topic of worldwide decline in sperm parameters is contentious (Mann et al., 2020). Manuscripts reported heterogeneous findings, with some studies confirming a decreasing trend in semen quality while others did not (Huang et al., 2017). A systematic review reported that sperm counts in western countries decreased by 50–60% between 1973 and 2011, and approximately one-third of cases remained idiopathic (Levine et al., 2017; Fainberg and Kashanian, 2019). Another study conducted a retrospective analysis of 119,972 men looking at total motile sperm count trends from 2002 to 2017 and revealed a decline of approximately 10% over the past 16 years (Tiegs et al., 2019; Mann et al., 2020). Jorgensen et al. (2012) showed an increasing trend in sperm concentration and total sperm count in 4,867 young men in Copenhagen, Denmark (Huang et al., 2017). Despite the decline in male fertility continues unabated, recent high-quality studies have demonstrated that there is indeed a decline in sperm parameters and have shown possible links for this decline in sperm parameters such as obesity, diet, and environmental toxins (Mann et al., 2020). Many efforts have been made to identify the deeper causes and develop new approaches to improve fertility in infertile men over the past few decades. However, there is currently few effective treatment to slow the decline in idiopathic male infertility.

The human microbiome is the focus of one of the most dynamic research fields of our time. The microbiome is a tiny ecosystem made up of thousands of species of microbes that mainly exist in external cavities, such as the reproductive tract, oral cavity, and gastrointestinal tract. Recent improvements in technology for collecting and analyzing DNA sequence data give a much deeper cognition of the diverse human microbiome. The complex composition of the human microbiome often poses challenges for scientists to classify and understand its effects on health. Studies in the microbiome field have found that the human microbiome plays a role in important physiological processes, such as host nutrient absorption immune system development, etc., and changes in human microbiome are closely related to the occurrence, development and treatment of a variety of diseases (Cani, 2018).

The “culture-negative” status of male reproductive tract samples in microbiological tests was once considered free of microbial infection, which has resulted in the male reproductive tract microbiota not being well described for many years. Yet, recent studies have found that a microbiota indeed exists in the male healthy reproductive tract and its body fluids, such seminal fluid and urine (Allen-Vercoe, 2013). As 16S ribosomal RNA sequencing has been applied to the microbiome of infertile males, correlations between human microbiome and semen parameters/fertility have been gradually revealed, which may be one of the important reasons why this disease has been so hard to track down in the clinic for so many years and is reluctantly called idiopathic male infertility (Lundy et al., 2020). Supplements of probiotics to modulate the microbiome have become a hotspot. It is well known that probiotics and their metabolites can alter the composition of the human microbiome and further influence the body’s metabolism and disease status. Considering that strategies using probiotics may benefit the microbial balance of the host, the influence of microbiota and probiotics on male infertility is gaining attention. Therefore, the potential of probiotics to affect the microbiome associated with male infertility has recently led to a significant increase in research interest.

Thus, the goal of this review was to give a current cognition of the microbiome’s potential effects on male infertility. In general, this review mainly included the following three aspects: the association between microbial infection in the genital tract and male infertility as well as the effect of antimicrobial therapy on male reproduction, the association between microbial dysbiosis and male infertility as well as the effect of probiotic intervention on male reproduction, and the limitations of current research as well as the suggestions for future research.

## Microbial Infection in the Genital Tract and Male Infertility

Studies devoted to understanding the role of microorganisms isolated in seminal fluid are conducted as early as 1976 (Kundsin et al., 1967; Busolo et al., 1984). The role of microbial infections in male infertility has been debatable (Huang et al., 2015). However, the impact of some microorganisms on male infertility has been extensively studied and established in the literature. Here, we will provide an overview of the relationship between microbial infections and male infertility. The relationship between bacterial infection, genital mycoplasmas or trachomatis infection, viral infection, and fungal infection with male infertility will be described in turn. In addition, we will present the effects of antimicrobial intervention on fertility in men with infection.

### Bacterial Infection Influences Male Infertility

*Escherichia coli* (*E. coli*) is among the most common pathogens having negative effects on male infertility (Berjis et al., 2018). *E. coli* is the most common cause of urogenital infection (Farsimadan and Motamedifar, 2020). Studies have provided compelling evidence indicating that *E. coli* infection has a detrimental effect on male fertility by reducing sperm motility or vitality and changing sperm morphology (Table 1). Semen quality in patients with *E. coli* infection can improve after the eradication of this infection, indirectly proving the detrimental role of *E. coli* in the genesis of male infertility (Vicari et al., 2016). As far as is known, the detrimental effect of *E. coli* on sperm may be via its adhesion to sperm and/or the expression of hemolysin A, a virulence factor involved in premature acrosomal activation and sperm nuclear DNA damage (Lang et al., 2013).

**TABLE 1:** Effects of genital microbial infection on sperm.

Types of genital microbial infection	Country, study subjects and size	PMID number of reference	Sperm concent- ration/count	Sperm motility	Normal sperm morphology	Anti-sperm antibodies	Sperm survival rate	Sperm volume	Sperm vitality	Sperm PH	Other parameters
*E. coli*	Iran Infertile men (*n* = 150) and healthy fertile controls (*n* = 150)	29997751		↓	↓						
*E. coli*	Chile Men with normozoospermic (*n* = 4)	19324341		↓					↓		Mitochondrial membrane potential↑, phosphatidylserine translocation↑, and ROS↑
*S. aureus*	Iran Infertile men (*n* = 150) and healthy fertile controls (*n* = 150)	30234189	↓	↓	↓			NA		NA	
*S. aureus*, *Neisseria gonorrhoeae*, *Enterococcus faecalis*, and *Gardnerella vaginalis*	Mexico Men attending an andrology clinic with negative bacteriological culture (*n* = 64) and positive bacteriological culture (*n* = 126) from semen samples	8554430	NA	↓	NA			NA	↓		
*S. aureus*, *E. coli*, and UUR	Poland Infertile men with genital tract infection (*n* = 39), and healthy controls (*n* = 30)	16112738	↓	↓	↓			↓	↓		
*S. aureus*, *Enterococcus faecalis*, and *Streptococcus agalactiae*	Germany Bacteriospermic men who need ICSI (*n* = 29), non-bacteriospermic men who need ICSI (*n* = 55)	29449095	↓	↓	NA						Sperm chromatin condensation↓, fertilization rate↓, and sperm protamine deficiency↑
*Enterococcus faecalis*	Italy Infertile men with negative microbiological testing (*n* = 190), positive microbiological testing (*n* = 83) from semen specimens	30444931		↓	↓				↓		
UUR	Wales Men with genital UUR infection (*n* = 140), men without genital UUR infection (*n* = 140)	6698224	↓	NA	NA	NA			NA		
UUR, *E. coli*, and *S. aureus*	Ireland Infertile men with genital tract infection (*n* = 39), infertile men without genital tract infection (*n* = 14), and healthy controls (*n* = 30)	16112738	↓	↓	↓			↓	↓		Rapid and slow progression of sperm cells (%)↓
UUR	United States Men with presumed chronic prostatitis with genital UUR infection (*n* = 17), men without genital UUR infection (*n* = 33), and healthy controls (*n* = 21)	10799180	↓	NA	NA						ROS↑, sperm fertilization capability↓
UUR, *C. trachomatis*, and *Mycoplasma* spp., etc	Italy Infertile men (*n* = 1689)	32299615	↓	↓	NA			NA			
UUR	China Infertile men with genital UUR infection (*n* = 31), infertile men without genital UUR infection (*n* = 23), and healthy controls (*n* = 27)	26856767	↓	↓	↓		↓			↓	Nitric oxide concentration↑, IL-17 and IL-18 concentrations↑, and sperm activation rate↓
*C. trachomatis*	Iran Infertile men (*n* = 165) and fertile controls (*n* = 165)	29292525	↓	↓	↓			NA			ROS↑, total antioxidant capacity↓
*C. trachomatis*	Finland Infertile men (*n* = 90) and fertile controls (*n* = 120)	18706556	↓	↓	NA	NA					IgG antibodies↑
*C. trachomatis*	Czechia Infertile men (*n* = 293)	21762193	↓								
*Mycoplasma* spp.	Czechia Infertile men (*n* = 293)	21762193	↓	↓	↓			↓			Sperm DNA fragmentation↑
*M. hominis*	Iran infertile men (*n* = 165) and fertile and healthy controls (*n* = 165)	27871827	↓	↓	↓			NA			ROS↑
*C. trachomatis*	Israel Infertile men (*n* = 175)	2298315	NA	NA	NA	↑					Sperm egg penetration ability↓
*Mycoplasmas*	Israel Infertile men (*n* = 175)	2298315	NA	NA	NA	↑					Sperm egg penetration ability ↓
*M. hominis*	Tunisia Infertile men (*n* = 120)	17988404	↓	NA	↓			NA	NA	NA	
UUR	Tunisia Infertile men (*n* = 120)	17988404	NA	NA	NA			NA	NA	NA	
HPV, HBV, etc.	United States Infertile men with leukocytospermia (*n* = 132) and infertile men without leukocytospermia (*n* = 109)	17433312	↓	↓							Neutral α-glucosidase concentration↓
HPV	China Infertile men (*n* = 615) and fertile controls (*n* = 523)	23603919	NA	↓	↓			NA			
HPV	China Men who attended the fertility clinics (*n* = 24)	9176459		↓							
HPV	Italy Men with genital warts (*n* = 26), men with HPV infection (*n* = 66), infertile men (*n* = 108), and fertile controls (*n* = 90)	20056213	↓	↓	NA			NA	NA	NA	
HPV	Italy Young adult men with previous sexual intercourse (*n* = 100) and young adult without previous sexual intercourse (*n* = 100)	19100537	NA	↓	NA			NA	NA	NA	
HBV and HCV	Iran Infertile men with HBV infection (*n* = 112), infertile men with HCV infection (*n* = 47), matched infertile controls with HBV negative and HCV negative (*n* = 112)	31583372	↓	↓	↓			NA		NA	
HCV	Egypt Men with HCV infection (*n* = 57), healthy controls (*n* = 40)	21620397	↓	↓	↓			↓			Prolactin↑, estradiol↑, and testosterone↓

*NA, not affected. Blank tables represent that the experiments are not conducted in this study.*

As a kind of symbiotic bacteria in human body, Staphylococcal species can attack reproductive tissues directly or through hematogenous pathway, and mediate the inflammatory response in reproductive system through innate immune pathway triggered by TLR2 (Dutta et al., 2020). Among Staphylococcal species, *Staphylococcus aureus* (*S. aureus*) is most closely related to male infertility. *S. aureus* may be an important adverse factor for deteriorating male reproductive function by decreasing sperm motility and increasing abnormal morphology, as well as decreasing sperm concentration (Esmailkhani et al., 2018). *S. aureus* along with other bacteria (*E. coli*, *UUR*, *Neisseria gonorrhoeae*, *Enterococcus faecalis*, *Gardnerella vaginalis*, and *Streptococcus agalactiae*) in male reproductive system is proved to be related to the lowering of sperm concentration, normal sperm morphology, sperm volume, and sperm vitality, as well as leads to the decreased fertilization rate, increased sperm protamine deficiency, and decreased sperm chromatin condensation (Table 1). In addition, high concentrations of *S. aureus* in seminal vesicles have been reported to be associated with sperm abnormalities (Esmailkhani et al., 2018). Also, *S. aureus* is able to immobilize and agglutinate spermatozoa, and thus colonization of *S. aureus* in male reproductive system should not be neglected in the treatment of male infertility (Dutta et al., 2020).

*Enterococcus faecalis* is found to negatively affect both sperm motility and morphology (Table 1). As mentioned in the previous paragraph, *Enterococcus faecalis* with other bacteria is proved to be related to the decreased semen quality.

In recent years, many other bacteria have been (e.g., *Enterococcus faecalis*, *Gardnerella vaginalis*, *Helicobacter pylori*, etc.) reported to be associated with male infertility, but independent studies on the relationship between single bacteria and male infertility are often lacking, so that we cannot tell which bacteria are really associated with male infertility (Merino et al., 1995; Gizzo et al., 2014; Moretti et al., 2017). Some studies have even drawn opposite conclusions about the relationship between the same bacteria and male infertility.

### Infection With Genital Mycoplasmas or Trachomatis Influences Male Infertility

Since 1967, *Ureaplasma* spp., infection in the male reproductive system has been considered as one of the causes of male infertility (Kundsin et al., 1967). It was reported that *Ureaplasma* was more frequently isolated from semen of infertile men (76%) than those of fertile men (19%) as early as 1974 (Friberg and Gnarpe, 1974). Since then, the correlation between *Ureaplasma* spp. infection and male reproductive function has been widely studied based on standard PCR based culture. In several recent studies, *Ureaplasma* spp. has been isolated from the semen of infertile men with frequencies ranging from 5 to 58% and from fertile men with frequencies ranging from 3 to 31% (Andrade-Rocha, 2003; Knox et al., 2003). Most previous studies did not divide *Ureaplasma* spp. into these two categories when discussing its role in male reproductive function: *Ureaplasma parvum* (UPA) and *Ureaplasma urealyticum* (UUR), among which the former comprises four serovars and the latter comprises ten serovars (Pitcher et al., 2001; Burrello et al., 2004). With further in-depth research, UUR has been shown to be a pathogen with a potential etiologic role in both genital infections and male infertility (Huang et al., 2015). Evidence suggests that a negative impact on sperm parameters, including sperm concentration, sperm motility, sperm volume, sperm vitality, normal sperm morphology, pH, and reactive oxygen species (ROS), was found in infertile men with UUR infection (Table 1). There are several possible pathophysiological mechanisms of UUR infection in infertile men, including direct pathogen-related DNA damage, membrane damage by cross-reactive antigens, or alterations in nitric oxide and interleukin-17/interleukin-18 expression (Shi et al., 2007; Gimenes et al., 2014; Qian et al., 2016). Nevertheless, UPA is more common than UUR in the semen of infertile and infertile men. Compared with UUR, UPA has higher pathogenicity and affects progressive movement and secretion function of semen (Zhou et al., 2018). Thus, the role of *Ureaplasma* spp. in the development of male infertility is beginning to be recognized.

*Chlamydia trachomatis* (*C. trachomatis*) is considered to be one of the most common sexually transmitted pathogens of the male reproductive tract (La Vignera et al., 2014). The detection rate of *C. trachomatis* in infertile men was several times higher than that in healthy fertile men (Ahmadi et al., 2018). Several studies agree with the negative impact of *C. trachomatis* infection on sperm count, sperm motility, normal sperm morphology, ROS production, total antioxidant capacity, and the egg penetration ability of sperm of infertile men (Table 1). Furthermore, *C. trachomatis* infection may lead to a higher incidence of male infertility by inducing the production of anti-sperm antibodies (Soffer et al., 1990).

*Mycoplasma hominis* (*M. hominis*) is also a common sexually transmitted pathogen with global distribution. The detection rate for *M. hominis* in infertile men was approximately more than three times higher than that in healthy fertile men (Huang et al., 2016; Ahmadi et al., 2017). A decrease in sperm count, motility, and rates of normal morphology, along with elevated ROS levels, was found in infertile men with *M. hominis* infection (Table 1). As a possible mechanism to explain the male infertility caused by *M. hominis* infection, Francisco Javier Diaz-Garcia et al. (2006) showed that *M. hominis* can adhere to the head, midpiece, or tail of the spermatozoa and can be internalized within cytosolic spaces (Diaz-Garcia et al., 2006).

### Viral Infection Influences Male Infertility

Human papillomavirus (HPV) is one of the well-known sexually transmitted viruses, which may lead to HPV-associated cancers in both men and women. Most studies on HPV have only focused on HPV-related diseases in women. Few data were available about HPV infection in men until a recent study showed the presence of HPV in semen (Wang et al., 2010). The findings have drawn intense attention to the effect of HPV infection on male fertility impairment (Gizzo et al., 2014). Lyu et al. (2017) showed that the pooled HPV prevalence was higher in fertility clinic attendees (20.4%, 95% CI = 16.2–24.6%) than in the general population (11.4%, 95% CI = 7.8–15.0%), providing clues to HPV prevalence in semen and its relationship with male reproductive function. HPV16 is a high-risk type of HPV commonly found in male anogenital sites, prostate, bladder, and oropharynx (Ndiaye et al., 2014; Yang et al., 2015). According to Lyu’s research, HPV16 is the most common type of HPV in semen, accounting for about 1/5 HPV-positive samples (Lyu et al., 2017). They also found that in semen, the positive rate of high-risk HPV56 was second only to that of HPV16. HPV56 is less common in HPV-related cancers (Lyu et al., 2017). Their conclusions are consistent with other evidence that HPV infection in semen may contribute to the risk of male infertility (La Vignera et al., 2015; Garolla et al., 2016). Baleful effects of HPV infection on male infertility have also been reported (Table 1), such as decreases in sperm count, sperm motility, sperm volume, and normal sperm morphology, which partly indicates that HPV infection is a risk factor for male infertility.

Impaired sperm parameters, including a decrease in semen volume, sperm count, and progressive sperm motility, as well as an increase in abnormal sperm morphology in male patients with hepatitis B virus (HBV)/hepatitis C virus (HCV) infection, have been found in several studies (Table 1). Exposure of human spermatozoa to HBV S protein enhances the early events of the apoptotic cascade and reduces sperm fertilizing capacity *in vitro*, which may explain the harmful effects of HBV on male infertility (J. Huang et al., 2013). Deleterious effects of HCV on male fertility are reflected not only by impaired sperm parameters but also by the lower total serum testosterone and higher serum E_2_ and prolactin levels than those of healthy controls (Hofny et al., 2011).

Although human immunodeficiency virus (HIV) can be detected in semen shortly after infection and during all subsequent stages of disease, current research suggests that sperm alterations can be attributed to the effects of anti-HIV therapy rather than HIV infection itself (Garolla et al., 2013; Liu et al., 2018). Nevertheless, other viruses, such as herpesviruses, cytomegalovirus, and adeno-associated virus, were also reported to be connected with male infertility; currently available evidence does not support a clear association between these factors and male fertility (Garolla et al., 2013).

### Fungal Infection Influences Male Infertility

*Candida albicans* (*C. albicans*) is one of the opportunistic pathogens of genitourinary infections, and its effect on male infertility has been poorly understood until a previous study reported that *C. albicans* is isolated from semen samples of asymptomatic men attending an infertility clinic (Rehewy et al., 1979). Then, the role of *C. albicans* on male infertility has been evaluated in several studies. *C. albicans* as well as its filtrates had an inhibitory effect on human sperm motility and impaired the ultrastructure of human spermatozoa *in vitro*, which could be associated with male infertility (Tian et al., 2007). Another study showed that the presence of *C. albicans* inhibited fertilization of oocytes and increased sperm DNA debris (Burrello et al., 2004). Fungal communities are likely closely tied to male infertility in ways we have only begun to characterize.

### Therapeutic Effects of Antimicrobial Agents on Male Reproductive Tract Infection

Asymptomatic infection, accompanied by recurrent and latent infections, is one of the major challenges in the treatment of infertility. Raising awareness on cleanliness of genital area among infertile patients and adopting appropriate means of infection prevention or control could be considered for the management of infertile men (Farsimadan and Motamedifar, 2020). Antibiotic therapy may be beneficial in maintaining or restoring normal sperm parameters in infertile men with infections and several classes of antibiotics may be used, such as quinolones, trimethoprim, tetracyclines. macrolides, β-lactam antibiotics, etc. Improvements in spermatozoal motility, including the speed of forward progression and the percentage of motile cells, were found in men who were successfully treated for UUR infection (Swenson et al., 1979). One study suggested that most semen parameters of male infertility associated with *C. trachomatis* infection returned to normal levels, and 42.9% of wives of *C. trachomatis*-infected infertile men became pregnant after antibiotic treatment (Ahmadi et al., 2018). Another study indicated that antibiotic therapy can improve semen parameters and treat male infertility induced by *Mycoplasma hominis*, resulting in 58.3% of female partners becoming pregnant after antibiotic therapy (Ahmadi et al., 2017). Although antibiotic treatment remains to be the cornerstone of treating bacterial/mycoplasma/chlamydia mediated infections, some antibiotics may damage sperm. Sperm damage has not been directly shown in humans in randomized clinical trials, but there is some data suggesting toxicity of certain antibiotics to testes and/or sperm in rats or mice, such as ciprofloxacin and pefloxacin, ofloxacin, lomefloxacin, tetracycline, cephalosporin and other cephalosporins, and norfloxacin (Calogero et al., 2017).

Few studies have been conducted to evaluate the efficacy of antiviral or antifungal therapy on sterile males with viral or fungal infections. Even still, researchers found that the treatment of HCV with antiviral drugs worsens semen parameters in sterile males (Hofer et al., 2010; Lorusso et al., 2010; Bukhari et al., 2018). More researches are needed to elucidate the pathologic mechanisms of the harmful effects of different microorganisms on the male reproductive system and how to stop microbial infection from damaging the male reproductive system, which will help to produce new treatments to save infertile men in the future.

## Microbial Dysbiosis and Male Infertility

Recent studies have suggested that the composition of the human microbiota is closely related to health outcomes (Natarajan and Bhatt, 2020; Tett et al., 2021). Since then, there is growing concern about the role of microbiota in reproductive health. Here, we will give an overview of the major findings and discuss the close relationship between microbiota and male infertility. Besides, we will also present some studies on the effects of probiotic supplementation on male reproduction.

### Gut Microbial Dysbiosis and Male Infertility

Most efforts on the microbiome are directed at the gastrointestinal tract, which harbors most of our microbes. Recent studies have found a direct relationship between dysbiosis of gut microbiota and male infertility, which may be an overlooked factor in clinical practice (Ding et al., 2020; Zhao et al., 2020; Lundy et al., 2021). It has been proven that the sperm concentration and motility were significantly decreased in mice treated with a high-fat diet, along with a decreased abundance of *Bacteroidetes* and *Verrucomicrobia* and an increased abundance of *Firmicutes* and *Proteobacteria* in their gut microbiota (Ding et al., 2020). Increased sperm concentration (twofold) and sperm motility (20-fold) were found after fecal microbiota transplantation from alginate oligosaccharide-dosed mice to busulfan-treated mice, and this was accompanied by an increase in the “beneficial” bacteria *Bacteroidales* and *Bifidobacteriales* (Zhang et al., 2021). Another relevant study suggested that alginate oligosaccharides can rescue busulfan-disrupted spermatogenesis in mice along with an increase in “beneficial” bacteria such as *Bacteroidales* and *Lactobacillaceae* and a decrease in “harmful” bacteria such as *Desulfovibrionaceae* in the gut microbiota (Zhao et al., 2020).

Although direct studies on the relationship of gut microbiota and male infertility are few, these current studies provide clues for further studies on the modification of gut microbiota in sterile male, which may provide valuable clues for treating male infertility.

### Microbial Dysbiosis in the Reproductive System and Male Infertility

Compared to microbiota in other body sites, the study on microbiota in the male reproductive system is relatively lacking. Previous studies have mainly focused on the detection of known pathogens based on traditional culture-dependent methods, targeted PCR amplification, and microscope observation. With the appearance of next-generation sequencing, it has become easier to determine the complex microbiota in the male reproductive system with high accurate and more detailed understanding of its interaction with male infertility.

A large number of studies have found that the disturbance of the female reproductive tract microbiota can lead to a series of reproductive diseases (Ravel et al., 2011; Phukan et al., 2013; Petrova et al., 2015). Although the social impact of reduced male fertility has been recognized, the male reproductive tract microbiota has been poorly studied and. As mentioned above, the “culture-negative” status of male reproductive tract samples in microbiological tests was once considered free of microbial infection, which has resulted in the male reproductive tract microbiota not being well described for many years. Yet, recent emerging evidence indicates that a microbiota indeed exists in the healthy reproductive tract and its body fluids, e.g., seminal fluid and urine (Allen-Vercoe, 2013). Microbiome investigations of male infertility focused on semen samples, and the results were relatively consistent for the microbiota in infertile men’s semen. Increased abundance of *Prevotella* and *Staphylococcus*, as well as decreased abundance of *Lactobacillus* and *Pseudomonas*, were shown in some studies (Weng et al., 2014; Baud et al., 2019; Farahani et al., 2021). Additionally, the abundance of *Prevotella* was negatively correlated with sperm motility and abnormal sperm morphology was directly correlated with the decreased abundance of *Lactobacillus* (Baud et al., 2019; Farahani et al., 2021).

Studies on the testicular microbiome are rare. Massimo Alfano et al. (2018) presented the first research evidence of a link between male infertility and alterations in the testicular microbiome. Compared with normal reproductive men, infertile men lacked *Bacteroidetes* and *Proteobacteria* in the testes. Tests with negative sperm retrieval at micro-testicular sperm extraction showed a decrease in *Firmicutes* and *Clostridium*, a complete lack of *Peptoniphilus asaccharolyticus*, and an increase in *Actinobacteria*. However, the fact that the control samples were from non-tumor regions of a tumor-bearing testis made it impossible to ignore the actual influence of the tumor microenvironment on the testicular microbiome. In addition, the small sample size of each group in this study further reduces its credibility. Lundy et al. (2021) found that both *Collinsella* (phylum *Actinobacteria*) and *Staphylococcus* (phylum *Firmicutes*) were depleted in semen after vasectomy, indirectly indicating the correlation between testicular microbiota and male infertility and providing further evidence for the relationship between male infertility and testicular microbiota. This study conducted by Lundy et al. (2021) also provided the first comprehensive survey of the rectum, semen, and urine microbiota of infertile men, in which similarities were found between the semen microbiota and the urine microbiota. Compared with the samples from fertile men, the rectum samples of infertile men showed decreased abundance of *Anaerococcus* and increased abundance of *Lachnospiraceae*, *Collinsella*, and *Coprococcus*; conversely, urine samples from infertile men contained increased *Anaerococcus*; semen samples of infertile men contained decreased *Collinsella* and increased *Aerococcus* (Lundy et al., 2021). Further study found a statistically negative correlation between *Aerococcus* abundance and both leukocytospermia and semen viscosity, a statistically negative correlation between *Prevotella* abundance and semen concentration, and a statistically direct correlation between *Pseudomonas* abundance and sperm count but inversely proportional to the pH of semen (Lundy et al., 2021).

Although the link between alterations in the male reproductive tract microbiota and male infertility is supported by the above studies, larger, multi-institution longitudinal studies are still needed to verify the accuracy of these results.

### Probiotic Supplementation for the Management of Male Infertility

With growing knowledge of the relationship between the microbial dysbiosis and male infertility, it is also necessary to make clear how probiotic supplementation affects male infertility. Here, we will show some outcomes of probiotic intervention in sterile males.

Probiotic supplementation has gained increasing attention as a treatment in various medical fields, including reproductive health, due to its low side effects. In recent years, the effect of probiotic supplementation on the human gastrointestinal microbiota has been fully demonstrated (Unno et al., 2015). Probiotic supplementation have also been studied during pregnancy, the treatment of bacterial vaginitis, and assisted reproduction technology (Larsson et al., 2008; Barbonetti et al., 2011; Hemalatha et al., 2012; Gille et al., 2016; Chenoll et al., 2019).

The first study on probiotics and human sperm was conducted *in vitro* and preliminarily proved the potential of a combination of three selected strains of *Lactobacilli* [*Lactobacillus brevis* (CD2), *Lactobacillus salivarius* (FV2), and *Lactobacillus plantarum* (FV9)] in protecting human spermatozoa from radical oxygen species in the presence of vaginal disorders, thereby improving the fertilization potential of the female host (Barbonetti et al., 2011). Studies using probiotic administration for male infertility showed that probiotics could be administered to improve sperm parameters and fertility-related endocrine parameters (Table 2). With the administration of probiotics (*Lactobacillus rhamnosus* CECT8361 + *Bifidobacterium longum* CECT7347), sperm quality parameters of asthenozoospermic males were statistically improved (Valcarce et al., 2017). As follows, significantly increased sperm motility (about sixfold variation), decreased DNA fragments (about 1.2-fold variation), and decreased intracellular H_2_O_2_ levels (about 3.5-fold variation) were found after probiotic administration (Valcarce et al., 2017). After treatment with Flortec (one sachet containing *Lactobacillus paracasei* B21060) in infertile men, many indicators that benefit male reproduction improved: volume of the ejaculate (median from 2.4 to 3.1 mL, *p* < 0.01), sperm concentration (median: from 15.2 × 10^6^/mL to 28.3 × 10^6^/mL, *p* < 0.01), progressive motility (median: from 16.2 to 42.0%, *p* < 0.01), and the percentage of typical forms (median: from 7 to 16.3%, *p* < 0.01) (Maretti and Cavallini, 2017). In addition, their follicle-stimulating hormone, luteinizing hormone, and testosterone also improved (*p* < 0.01) after treatment with Flortec (Maretti and Cavallini, 2017). No modification was found in infertile men treated with control substances (starch) (Maretti and Cavallini, 2017). In another study on infertile men, their ejaculate volume (mean from 3.6 ± 0.91 mL to 4.94 ± 0.63 mL, *p* = 0.041), total sperm count (mean from 57.6 ± 7.09 10^6^ sperm/ejaculate to 79.04 ± 14.21 sperm/ejaculate, *p* = 0.001), sperm concentration (mean from 16.25 ± 4.5 × 10^6^/mL to 20.77 ± 6.49 × 10^6^/mL, *p* = 0.001), percentage of motile sperm (mean from 25.19 ± 6.46% to 33.21 ± 7.91%, *p* = 0.037), live sperm (mean from 52.37 ± 10.13 to 62.43 ± 12.17, *p* = 0.003), and serum and seminal total antioxidant capacity (mean from 1.67 ± 0.19 μmol/L to 2.33 ± 0.6 μmol/L, *p* < 0.001) significantly increased; the serum and seminal malondialdehyde (mean from 0.9 ± 0.11 μmol/L to 0.69 ± 0.07 μmol/L, *p* = 0.003) and plasma inflammatory markers (mean of CRP from 6.93 ± 2.11 μM to 4.01 ± 1.09 μM, *p* = 0.001; mean of TNFα from 11.28 ± 3.12 μM to 8.85 ± 2.49 μM, *p* = 0.003) significantly decreased after intervention with *Lactobacillus* and *Bifidobacteria* species (Helli et al., 2020).

**TABLE 2:** Intervention of probiotics on males and their sperm.

Probiotics	Study subjects and size	PMID number of reference	Sperm motility	Sperm count	Normal sperm morphology	Sperm concentration	Sperm volume	Sperm viability	Antioxidant capacity	Sex hormones	Inflammatory factors	Others
*Lactobacillus brevis* [CD2], *L. salivarius* [FV2], and *L. plantarum* [FV9]	Normozoospermic men who sought medical care for infertility (*n* = 10)	21497805	↑					↑	↑			
*Lactobacillus rhamnosus* CECT8361 and *Bifidobacterium longum* CECT7347	Asthenozoospermic men without any medical treatment (*n* = 9)	28343402	↑					NA				Sperm DNA fragmentation↑
*Lactobacillus paracasei* B21060	Oligoasthenotera- tospermia men (*n* = 20)	28245352	↑	↑	↑	↑	↑			↑		
*Lactobacillus casei*, *Lactobacillus rhamnosus*, *Lactobacillus bulgaricus*, *Lactobacillus acidophilus*, *Bifidobacterium breve*, *Bifidobacterium longum*, and *Streptococcus thermophiles*	Men with idiopathic oligoasthenotera- tozoospermia (*n* = 52)	32985280	↑	↑		↑	↑		↑	NA	↓	
*Bacillus subtilis* KA TMIRA1933 and *Bacillus amyloliquefaciens* B-1895	Male chickens (*n* = 20)	29238921			↑	↑						
*Lactobacillus coagulans* and *Lactobacillus casei*	Male Wistar rats (*n* = 32)	33225493		↑	↑			↑	↑	↑		Benefit parameters of testis↑
*Lactobacillus rhamnosus Gorbach–Goldin* (LGG)	Kunming mice (*n* = 18)	32265160	↑	↑	↑				↑	↑	↓	Sperm DNA fragmentation↑
*Lactobacillus rhamnosus* PB01 (DSM-14870)	Male C57BL/6N Tac mice (*n* = 24)	29016685	↑							↑		
*C. utilis* (Cu.M02) and *S. thermophilus* (St.S07)	Male ICR mice (*n* = 75)	22207218	↑	↑	↑					↑		
*Lactobacillus rhamnosus* CECT8361 and *Bifidobacterium longum* CECT7347	Adult zebrafish males (*n* = 36)	31013929	↑			↑						

*NA, not affected. Blank tables represent that the experiments are not conducted in this study.*

In addition, treatment with probiotics in an animal model was also proven to improve sperm parameters, antioxidant capacity and fertility-related endocrine parameters. The use of probiotics based on either *Bacillus subtilis* KA TMIRA1933 and *Bacillus amyloliquefaciens* B-1895 or of a mixture of strains in pedigree roosters can effectively improve the quality of sperm production, including increasing the volume of ejaculate, the spermatozoa concentration, and the total number of spermatozoa in the ejaculate, as well as decreasing the number of morphologically abnormal semen cells (Mazanko et al., 2018). The ameliorative effects of probiotics on sperm parameters and reproductive hormone levels have also been reported in different male murine models (Ibrahim et al., 2012; Dardmeh et al., 2017; Guo et al., 2020; Helli et al., 2020; Keshtmand et al., 2021). These probiotics, including *Candida utilis* (Cu. M02) and *Streptococcus thermophilus* (St. S07), *Lactobacillus coagulans* and *Lactobacillus casei*, *Lactobacillus* and *Bifidobacteria* species, and *Lactobacillus rhamnosus* PB01 (DSM 14870) (Ibrahim et al., 2012; Dardmeh et al., 2017; Guo et al., 2020; Helli et al., 2020; Keshtmand et al., 2021).

In addition to the effects on roosters and murine models, probiotics have also been investigated for possible male reproductive impacts in zebrafish models. Feeding zebrafish with *Lactobacillus rhamnosus* CECT8361 and *Bifidobacterium longum* CECT7347 positively increased their sperm concentration, total motility, progressive motility, and fast spermatozoa subpopulations (Valcarce et al., 2019).

Therefore, oral intake of probiotics has the potential to be one of the ways to address male infertility. The main features and results obtained in studies involving probiotics and male infertility are shown in Table 2.

## Limitations of Current Studies and Future Research Trends

### Limitations of the Current Studies on Microbiome and Male Infertility

Although many studies in this review are labeled randomized controlled trials, the randomization methods were not described in detail, the vast majority of controls were lacking or were blank controls, and almost no double-blind clinical studies were available, which greatly reduces the effectiveness of the research evidence. However, the accurate evaluation of the microbiome in male infertility is exceptionally difficult because of many confounding factors, such as age, body mass index, drinking and smoking habits, diet profile, scale and term of study, and medication exposure, may also affect microbiome and reproductive parameters. Standardization remains a critical issue hampering the implementation of microbiome analysis in clinical practice. Microbiome analysis may yield questionable conclusions of relevance to human disease if basic research information between cases and controls does not match (Vujkovic-Cvijin et al., 2020). This review is also limited by the small sample sizes in each study, application of different animal models, heterogeneity of the study types, discordance of reported outcomes, large variation in antimicrobial drug or probiotic administration, and limitation of sequencing range of 16S rRNA technology. Most studies have not examined the effect of the microbiome on clinical fertility outcomes and progeny of infertile men, such as clinical pregnancy rate and live birth rate.

Given the lack of evidence in the literature on this topic, further large-cohort prospective studies are needed to corroborate the importance of the microbiome as a cause of male infertility. Nonetheless, further work exploring the deeper mechanisms, as well as appropriate criteria for identifying patients with microbiome-induced infertility is required.

### Future Trends in the Microbiome Association With Male Infertility

Although microbial infection or dysbiosis can affect male infertility, to date, only scant information is available about the influence of microbial infection or dysbiosis or its exact molecular mechanisms. Research in this field is just getting started, and there is still much work to be done in this area. For example, authoritative studies in this direction are expected to find out whether antibiotics cure infertile men by eliminating microbes, or whether there are other mechanisms involved, such as infection of the body’s microbiome or immune system. Also, the study of female infertility and microbiota may have many implications for us.

Given the lack of research on the relationship between the microbiome and male infertility, the three steps of correlations, causality, and mechanisms need to be fully explored traditionally. Specifically, correlation analysis mainly involves sequencing samples from different sources to analyze the changes in microbiome structure, abundance, and species between infertile men and fertile men/cured infertile men. In the study of causality, methods of fecal microbiota transplantation or probiotic supplementation are often used to study the relationship between the microbiome and diseases from a macroscopic perspective. Mechanistic exploration is based on multiomics methods (metagenomics, transcriptomics and metabolomics, etc.) to identify differential metabolites and targets for further intervention. The rapid development of next-generation sequencing will give us a more detailed understanding of the microbiome in infertile men. Subsequent work must use a large number of samples from multiple sampling centers in order to gain a clear understanding of the microbiome and its changes during adolescence, first sexual activity, adulthood, and old age, as well as its impact on reproductive function and reproductive disease processes (Farahani et al., 2021). Nevertheless, there is also room for bold research innovations in this area, and integrated studies will thereby allow us to truly advance research on the microbiome and male infertility, moving from association to modulation. In the process of studying microbiome and male infertility, factors such as genetic mutations, diet, environmental factors, drugs, female infertility, fertility outcome, and mental condition can be included to make the research results more comprehensive and convincing. Therefore, the design of future studies should attempt to eliminate confounding factors in order to better understand the relationship and regulatory mechanisms between microbiota and infertile male (Bordalo Tonucci et al., 2017). Each study should provide a comprehensive description of its entire protocol and procedure for microbiome assessment, including experimental design, sequencing platform, sequencing area and applied database, to enhance the usefulness of these studies to other investigators. Therefore, it is recommended that future studies in this area should be conducted in a multicentre, randomized controlled trial or with a randomized double-blind placebo-controlled design. In addition, future studies should also focus on non-pathogenic organisms that may have a protective role, and how these organisms can be developed as therapeutic options (e.g., probiotics), and attention should be especially paid to the safety monitoring of probiotics in sterile males. The key process in studying the microbiome of infertile males is from understanding correlation to determining causal molecular mechanisms. We are entering an era of personalized microbiome medicine, where treatments can be tailored not only to human genetic polymorphisms but also to the make-up of individual microbes.

## Conclusion

There is growing evidence that an imbalance of the human microbiome can cause a range of diseases, some of which can be treated by restoring the symbiotic microbiome. Breakthrough research on the microbiome has opened up new ideas for disease treatment, and microbiome therapy has emerged. This review provides a new perspective for the prevention and treatment of male infertility, especially the previously unknown etiology of idiopathic male infertility, and proposes a new idea for its precise treatment with probiotics. With increasing evidence of the relationship between the human microbiome and various diseases, the use of these results to prevent, treat, and predict the prognosis of diseases has become a research hotspot. Research on the microbiome of sterile males can provide a more comprehensive understanding of the pathogenesis of male infertility, and individualized treatment of the microbiome can be carried out to meet the fertility needs of sterile males.

## Author Contributions

XL designed the review, wrote the manuscript, performed the literature research, and drafted the manuscript. HW and AX performed revisions and critically discussed the complete manuscript. LG was responsible for the concept and final revision of the manuscript. BZ and ZC participated in the content design, data supplement, and manuscript modification during the process of revising this review. All authors contributed to the article and approved the submitted version.

## Conflict of Interest

The authors declare that the research was conducted in the absence of any commercial or financial relationships that could be construed as a potential conflict of interest.

## Publisher’s Note

All claims expressed in this article are solely those of the authors and do not necessarily represent those of their affiliated organizations, or those of the publisher, the editors and the reviewers. Any product that may be evaluated in this article, or claim that may be made by its manufacturer, is not guaranteed or endorsed by the publisher.

## Abbreviations

UPA: *Ureaplasma parvum*
UUR: *Ureaplasma urealyticum*
*C. trachomatis*: *Chlamydia trachomatis*
*M. hominis*: *Mycoplasma hominis*
HPV: human papillomavirus
HBV: hepatitis B virus
HCV: hepatitis C virus
HIV: human immunodeficiency virus
*E. coli*: *Escherichia coli.*
